# StarD5 Plays a Critical Role in the Hepatocyte ER Stress Survival Response

**DOI:** 10.3390/ijms26094157

**Published:** 2025-04-27

**Authors:** William M. Pandak, Genta Kakiyama, Daniel Rodriguez-Agudo

**Affiliations:** 1 Department of Internal Medicine, School of Medicine, Virginia Commonwealth University, Richmond, VA 23298, USA; william.pandak@va.gov (W.M.P.); genta.kakiyama@vcuhealth.org (G.K.); 2 Central Virginia VA Healthcare System, Richmond, VA 23248, USA

**Keywords:** endoplasmic reticulum stress, unfolded protein response, StarD5, liver steatosis, apoptosis

## Abstract

The unfolded protein response (UPR) is a highly orchestrated survival response initiated in cells under endoplasmic reticulum (ER) stress. Steroidogenic acute regulatory-related lipid transfer domain 5 (StarD5) is an ER stress-responsive, cholesterol-binding protein under the regulation of IRE1. Based upon in vitro findings, StarD5 delivers a protective response by translocating ER cholesterol to the plasma membrane (PM) and accompanying protective changes in PM fluidity. The study aimed to determine if StarD5′s ability to provide in vitro hepatocyte protective responses is translatable to in vivo conditions. ER stress in mouse livers was induced by intraperitoneal injection of tunicamycin (Tm). Adenovirus was used to restore the expression of StarD5 in the livers of StarD5^−/−^ mice. Immunoblotting, histological analysis, and lipid measurements were performed. Induction of ER stress led to increased expression of StarD5 and steatosis in the livers of wild-type (WT) mice, while in StarD5^−/−^ mice, steatosis and apoptosis were more acute compared to WT mice, as evidenced by increased lipid accumulation and cleavage of PARP, respectively. Selectively restoring StarD5 expression to ER-stressed StarD5^−/−^ mice blunted the effects of tunicamycin. StarD5 appears to play a critical role in the ER stress survival response through its ability to regulate intracellular cholesterol homeostasis.

## 1. Introduction

The endoplasmic reticulum (ER) is the primary subcellular compartment responsible for the synthesis, folding, and maturation of secreted and transmembrane proteins in cells [1]. The proper functionality of the ER is essential for cell survival and is highly sensitive to changes in overall cell homeostasis [2]. Various conditions can induce cellular stress, leading to alterations of homeostasis within the ER, which can cause the accumulation of misfolded or unfolded proteins in the ER lumen [1,3]. The accumulation of unfolded proteins leads to the activation of the ER stress response known as the unfolded protein response (UPR) [4]. Activation of the UPR depends on three ER transmembrane proteins—activating transcription factor 6 (ATF6), inositol-requiring enzyme I (IRE1), and RNA-activated protein kinase-like ER kinase (PERK)—and one master regulator that functions as an ER chaperone protein known as binding immunoglobulin protein (BiP; also known as glucose-regulated protein 78/GRP78) [5]. Under normal conditions, BiP binds to the luminal domains of ATF6, IRE1, and PERK, preventing their activation. In response to ER stress, BiP is released from ATF6, IRE1, and PERK, and binds to unfolded or misfolded proteins in the ER, resulting in the activation of the three stress transducers [6], inducing the UPR response [4,5,6,7]. In addition to being the main compartment for the synthesis of proteins, the ER is also the main site of cholesterol synthesis, with synthesized cholesterol rapidly transported to other organelles [8]. Overaccumulation of free cholesterol in the ER is one of the physiological stress conditions that induces the UPR [9]. During ER stress, cholesterol buildup initiates the UPR, in which proteins like steroidogenic acute regulatory-related lipid transfer domain 5 (StarD5) are essential for cholesterol transport and maintaining homeostasis. StarD5 is a cholesterol-binding protein that mediates the translocation of cholesterol from the ER to the plasma membrane (PM) and is regulated under ER stress by RE1 [10,11]. Given that the expression of StarD5 is regulated in response to ER stress, we hypothesized that ER stress leads to increased StarD5 expression and redistribution of the StarD5 protein to subcellular locations where it is needed under conditions of ER stress (i.e., the cell membranes). Based upon previous in vitro findings, we speculated that the increased expression of StarD5, as well as its subcellular redistribution, plays a protective role by removing cholesterol from the ER, thereby reducing the cholesterol levels in the ER during the cell’s protective phase of ER stress.

In the present study, we used a StarD5 knockout mouse line to investigate the in vivo role of StarD5 in intracellular cholesterol distribution and homeostasis, after eliciting steatosis by inhibiting post-translational modification N-linked glycosylation of proteins in the ER using the drug tunicamycin (Tm) [12,13]. The results demonstrate a correlation between the presence of StarD5 protein and the ability of the mouse liver to protect itself from ER stress, as evidenced by reduced programmed cell death, i.e., apoptosis.

## 2. Results

### 2.1. Tunicamycin Injection Induces StarD5 Expression in Livers

**Figure 1:**
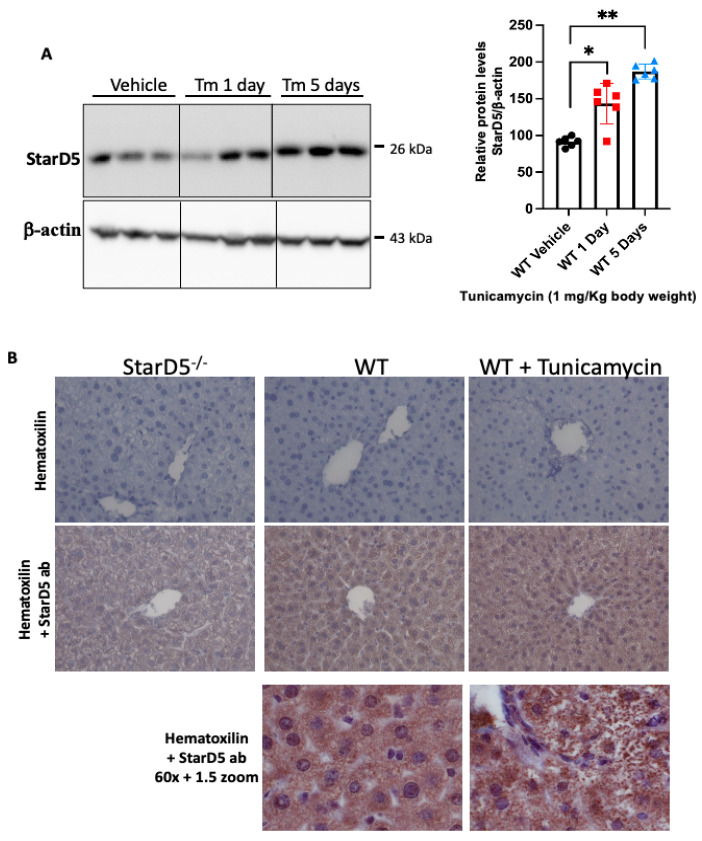
Tunicamycin-induced ER stress in vivo increases StarD5 expression in the liver. (**A**) Representative immunoblots of StarD5 and β-actin (as loading control) were performed on liver homogenates from WT and StarD5^−/−^ mice injected with vehicle or tunicamycin for 1 or 5 days. Relative expression levels were determined by densitometry of StarD5 relative to GAPDH. n = 6. * *p* < 0.05. ** *p* < 0.01. (**B**) Sections from WT and StarD5^−/−^ mouse livers were immunostained for the detection of StarD5 and stained with hematoxilin. Images were visualized using 20× objective and 60× objective. Representative images from three mice are shown. Note the increased staining following tunicamycin injection after 5 days.

We and others have shown that ER stress can induce StarD5 expression in cells [10,11,14]. To induce ER stress in vivo in the liver, we used tunicamycin because it has few off-target effects, elicits a robust response, predominantly targets the liver and kidney, and is not lethal in wild-type mice even at high doses [13,15,16]. Following peritoneal injection of tunicamycin, StarD5 expression increased significantly in livers at 1 and 5 days after the injection (Figure 1A). In addition, immunohistochemistry studies were performed on liver samples from WT mice injected with vehicle or tunicamycin. Livers from WT mice showed staining for StarD5, while those from StarD5^−/−^ mice did not (Figure 1B). Following tunicamycin injection, the pattern of StarD5 staining in cells within the liver tissue also changed (Figure 1B).

### 2.2. Tunicamycin-Induced ER Stress In Vivo Leads to Weight Loss While Increasing Lipid Accumulation in Livers

**Figure 2:**
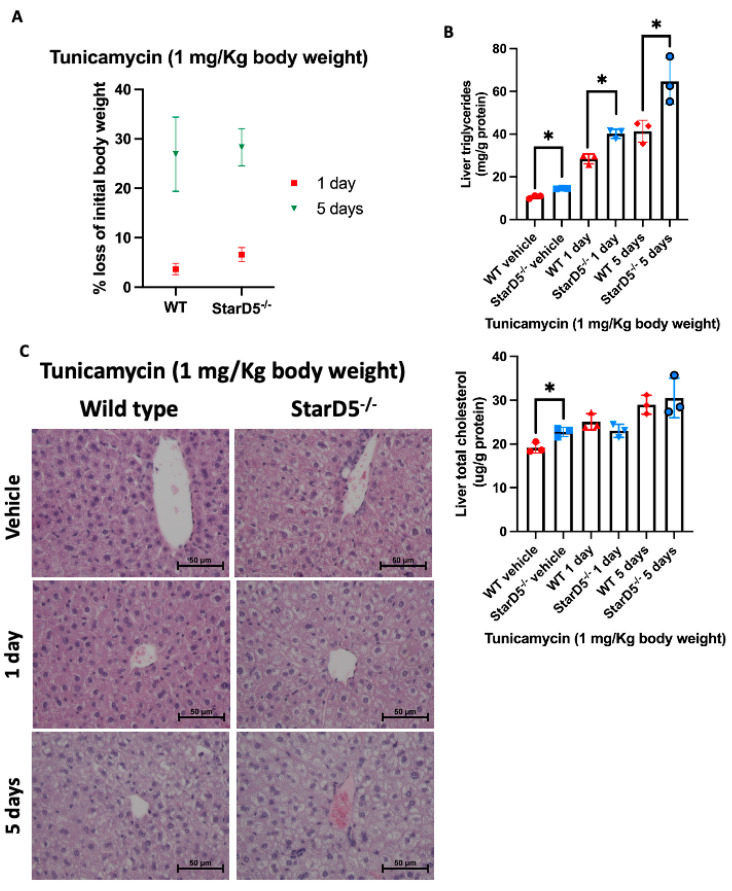
Tunicamycin-induced ER stress in vivo leads to weight loss and increases lipid accumulation in the livers of StarD5^−/−^ mice compared to wild type. (**A**) Percentage of body weight loss in WT and StarD5^−/−^ mice injected with tunicamycin for 1 or 5 days; n = 3. (**B**) Total cholesterol and triglycerides were determined in livers of WT and StarD5^−/−^ mice injected with vehicle or tunicamycin for 1 or 5 days. n = 3. * *p* < 0.05. (**C**) Sections from WT and StarD5^−/−^ mouse livers were stained with H&E and visualized using a 40× objective. Representative images from three mice are shown. Note the extensive cytoplasmic vacuolization in the livers from StarD5^−/−^ animals. n = 3.

Chemically induced ER stress has been reported to cause weight loss and anorexia [13,17]. This was also the case in our mouse studies, where injection of tunicamycin led to weight loss, which was slightly greater in StarD5^−/−^ mice compared to WT mice, though not significantly different (Figure 2A). Interestingly, when lipid content was analyzed in the livers of both WT and StarD5^−/−^ mice, we observed an increase in triglyceride content following tunicamycin injection. This increase was significantly greater in StarD5^−/−^ mice compared to WT mice at either 1 or 5 days post-injection (Figure 2B). This observation is consistent with findings we have described previously, in which triglyceride liver content in StarD5^−/−^ mice was found to be elevated compared to WT mice under normal conditions [11,18]. Conversely, we found that the level of cholesterol in the livers was not significantly different between WT and StarD5^−/−^ mice at either day 1 or 5 following the tunicamycin injection (Figure 2B). Histological analysis (H&E staining) was also performed on liver samples from WT and StarD5^−/−^ mice with or without tunicamycin injection (Figure 2C). As previously shown [11], ablation of StarD5 leads to an accumulation of lipid droplets compared to WT mice (top panels of Figure 2C). At both 1 and 5 days following the injection of tunicamycin, WT and StarD5^−/−^ livers presented an increased vacuolization, more pronounced in StarD5^−/−^ livers (central and bottom panels of Figure 2C), which corresponds with the increased triglyceride levels observed in the biochemical analysis of these livers (Figure 2B). When analyzing key enzymes in the cholesterol and triglyceride synthesis pathways (HMGCR and FAS, respectively), immunoblots revealed that HMGCR did not change (Supplemental Figure S1), aligning with the minimal change in cholesterol content (Figure 2B) upon tunicamycin injection. Interestingly, FAS expression was significantly reduced following tunicamycin injection in all cases (Supplemental Figure S1), an indication that the triglycerides being accumulated in the liver following tunicamycin injection are not coming from newly synthesized fatty acids.

### 2.3. Tunicamycin-Induced ER Stress In Vivo Leads to an Increase in Apoptosis in Livers of StarD5^−/−^ Mice Compared to Wild Type Livers

**Figure 3:**
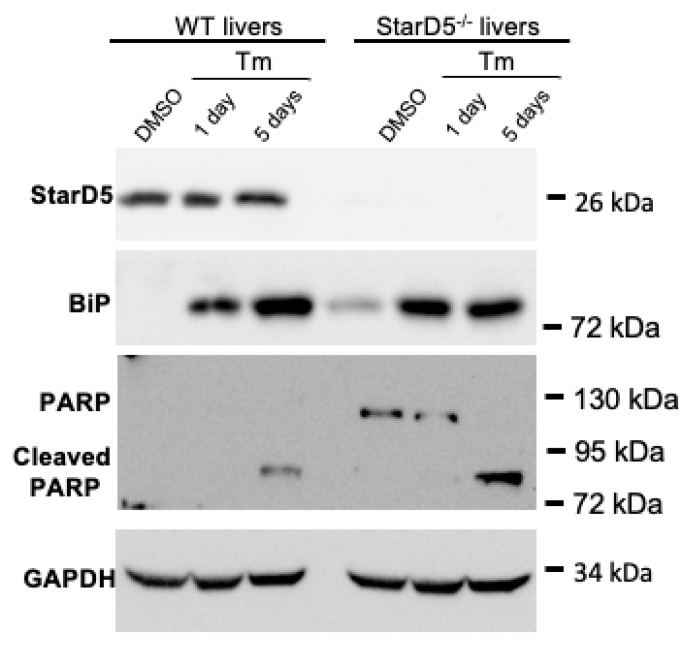
Tunicamycin-induced ER stress in vivo increases apoptosis in livers of StarD5^−/−^ mice compared to wild type livers. Representative immunoblots of StarD5, BiP, PARP, and GAPDH (loading control) in livers of WT and StarD5^−/−^ mice injected with vehicle or tunicamycin (Tm) for 1 or 5 days.

Initial activation of the ER stress response is well characterized by increased expression of GRP78/BiP [19,20], as seen in the livers of WT and StarD5^−/−^ mice following tunicamycin injection (Figure 3). We further explored the activation of the ER stress pathway to determine apoptosis. For that, we analyzed the expression of the apoptotic marker PARP [21]. Cleavage of PARP is indicative of cells being driven to apoptosis. As shown in Figure 3 and Supplemental Figure S1A, the injection of tunicamycin did not lead to the cleavage of PARP after 1 day, but cleavage was evident in both WT and StarD5^−/−^ mice after 5 days, with greater levels in the livers of StarD5^−/−^ mice compared to WT mice (Figure 3 and Supplemental Figure S1B). This indicates that the absence of StarD5 leads to greater susceptibility to apoptosis under ER stress conditions.

### 2.4. Restoring Expression of StarD5 Reduces ER Stress and Apoptosis in StarD5^−/−^ Mice

**Figure 4:**
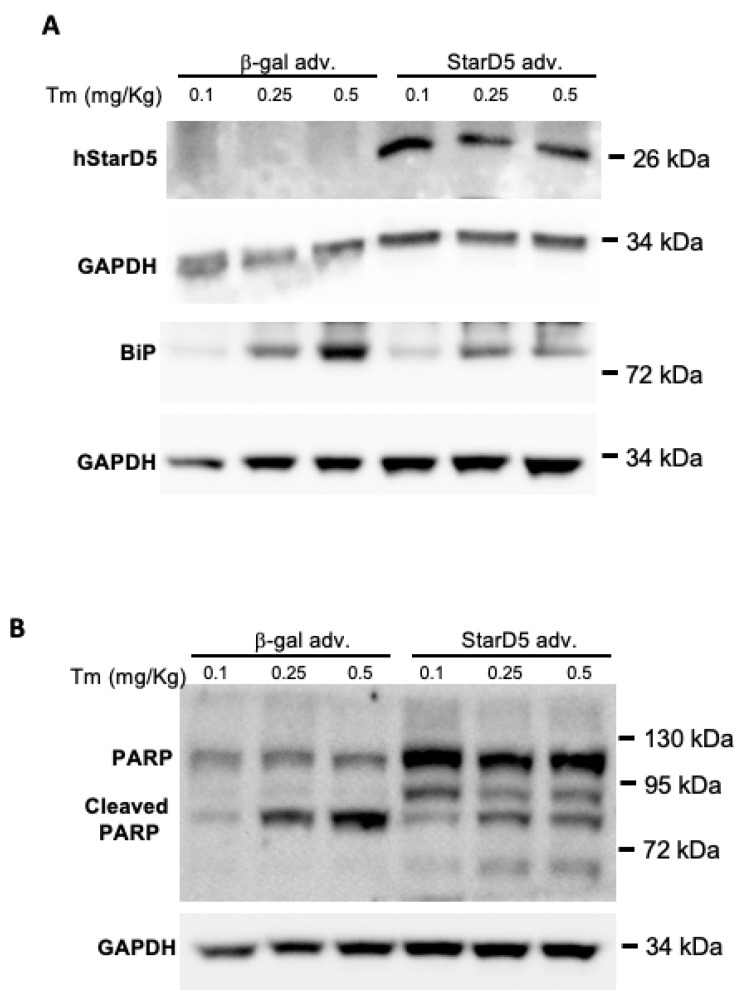
StarD5 overexpression in StarD5^−/−^ mice reduces apoptosis following tunicamycin-induced ER stress in vivo. StarD5^−/−^ mice were injected with an adenovirus encoding either StarD5 or β-gal (control) to overexpress StarD5, and after a week injected with different amounts of tunicamycin (Tm) to induce ER stress. Immunoblots of StarD5 and BiP (**A**) and PARP (**B**) with GAPDH (as loading control) in livers of WT and StarD5^−/−^ mice were obtained.

To determine if selectively restoring the expression of StarD5 in the livers of StarD5^−/−^ mice could reduce hepatic ER stress and apoptosis, we performed adenovirus-mediated StarD5 overexpression in StarD5^−/−^ mice followed by injection of different doses of tunicamycin. The mice were injected with either Ad-CMV-hStarD5.MycDDK or control Ad-CMV-β-gal, and later with tunicamycin, as described in the Materials and Methods section. Five days after the tunicamycin injection, the mice were euthanized and their livers collected for analysis. As shown in Figure 4A, all mice that received the Ad-CMV-hStarD5.MycDDK expressed StarD5 in the liver, while those that received Ad-CMV-β-gal did not express StarD5. In addition, mice that received the Ad-CMV-β-gal saw an increased expression of BiP in a dose-dependent manner relative to the amount of tunicamycin injected, but those mice that received the Ad-CMV-hStarD5.MycDDK saw a blunted expression of BiP compared to the controls with Ad-CMV-β-gal (Figure 4A). Finally, the samples were analyzed to determine whether restoring the expression of StarD5 could reduce apoptosis. Indeed, following upregulation of expression of StarD5, the levels of cleaved PARP were reduced in all three different doses of tunicamycin compared to the mice that were injected with Ad-CMV-β-gal (Figure 4B). These results indicate that restoring StarD5 can ameliorate both ER stress and apoptosis under the conditions described.

## 3. Discussion

Triggering in vivo ER stress in mice led to a significant increase in the expression of StarD5 in livers of WT mice 1 and 5 days after peritoneal injection of tunicamycin (Figure 1A). This was a predicted response based on multiple (macrophage and hepatocyte) in vitro studies showing regulation of StarD5 by ER stress [10,14], and recent in vivo findings in mice fed a Western diet [18]. In addition to StarD5′s increase in expression following the tunicamycin injection (Figure 1B), the change in the localization of StarD5 within the cell with onset of the ER stress appears to represent its redistribution as StarD5 attempts to facilitate cholesterol movement between membranes as described previously [11].

The deletion of StarD5 had a significant effect on ER stress, as evidenced by its effects on apoptosis. As shown in Figure 3 and Supplemental Figure S1B, the absence of StarD5 markedly increased the cleavage of the apoptotic marker PARP 5 days post tunicamycin injection. It should also be noted that the presence of full-length PARP in StarD5^−/−^ samples, but not in WT samples (with DMSO and 1 day after tunicamycin injection), may be explained by the previously described activation in fatty liver [22,23], as it happens in StarD5^−/−^ mice under normal conditions (Figure 2C top panel) and WT (5 days) and StarD5^−/−^ livers following tunicamycin injection. To confirm the effect, mitigated by the selective absence of StarD5, we performed rescue experiments by restoring StarD5 expression in StarD5^−/−^ mice (Figure 4A). When comparing rescued StarD5^−/−^ mice with control mice (StarD5^−/−^) receiving a β-gal adv, mice with restored expression of StarD5 showed a significant decrease in ER stress as measured by BiP expression, as well as decreased cleavage of the apoptosis marker PARP (Figure 4A,B). The effects of restoring StarD5 clearly made the liver more resilient to unmitigated ER stress and apoptosis.

A common effect of the tunicamycin injection was the loss in body weight in both WT and StarD5^−/−^ mice (Figure 2A). This effect has been described as anorexia-induced lipolysis [17]. Unmitigated ER stress is known to lead to lipolysis in different tissues in the mice and to steatosis in the liver and kidney. The hepatic steatosis was clearly present in all mice, as evidenced by the vacuolization of cells in liver samples of WT and StarD5^−/−^ mice (Figure 2C). Biochemical analysis of cholesterol and lipids within the livers of these mice did not show a significant increase in cholesterol. Triglycerides, however, were significantly elevated following tunicamycin injection at 1 and 5 days (Figure 2B), with StarD5^−/−^ mice significantly higher than WT mice. The unchanged levels of cholesterol and the rate-determining enzyme of cholesterol synthesis, HMGCR (Supplemental Figure S1A,B), paralleled each other, as previously described in a similar model where SERBF2 levels were also unchanged [17]. In contrast, fatty acid synthase (FAS) was clearly suppressed under ER stress conditions in the liver (Supplemental Figure S1A,B), while triglycerides were increased (Figure 2B). This disparity between unchanged cholesterol levels and elevated triglycerides in the liver has been explained by the relocation of triglycerides from peripheral tissues into the liver, as has been shown previously [17]. Interestingly, when comparing triglyceride levels in the livers of WT and StarD5^−/−^ mice, steatosis in StarD5^−/−^ mice was clearly more prominent (Figure 2B,C). We recently demonstrated that VLDL secretion is reduced in the absence of StarD5, likely contributing to the increased liver triglyceride content in StarD5^−/−^ mice, making StarD5^−/−^ mice more prone to developing steatosis and fibrosis [18].

## 4. Materials and Methods

The Amplex Red Cholesterol and Infinity Triglycerides assay kits and SuperSignal West Pico Chemiluminiscent Substrate were obtained from Thermo-Fisher Scientific (Waltham, MA, USA). Antibodies used in the study are listed in Table 1. Ad-CMV-hStarD5.MycDDK and Ad-CMV-β-gal were prepared by SignaGen Laboratories (Frederick, MD, USA) using a plasmid encoding human StarD5 cDNA fused to myc and flag tags, driven by the CMV promoter (Catalog # RC202407), which was from Origene (Rockville, MD, USA). Multiplicity of infection (MOI) was quantified with the Adeno-X Rapid Test Kit from Takara-Bio (Cat#632270, San Jose, CA, USA).

### 4.1. Animal Studies

All animal studies and care were approved (ethics approval number 01756) and conducted under the guidelines of the Virginia Commonwealth University (VCU) and the Central Virginia VA Healthcare System Institutional Animal Care and Use Committees (IACUC/D16-00688), in accordance with the principles and procedures outlined in the National Research Council’s *Guide for the Care and Use of Laboratory Animals* (Assurance Number A3281-01). Animals were housed individually in ventilated cages within a barrier vivarium, which excludes all known mouse viruses, parasites, and most bacteria (including helicobacter), and were fed standard mouse chow (irradiated Teklad LM-485 diet; Inotiv, West Lafayette, IN, USA) and autoclaved water.

### 4.2. Generation of StarD5 Knockout (StarD5^−/−^) Mice, Preparation and Injection of Tunicamycin Solution

StarD5^−/−^ mice were generated using a CRISPR/Cas9 approach as previously described [11]. Tunicamycin was prepared in DMSO at 5 mg/mL, flash-frozen in 25 mL single-use aliquots, and stored at −20 °C. Wild-type (C57BL/6) and StarD5^−/−^ mice were injected intraperitoneally with tunicamycin at 1 mg/kg, diluted in sterile PBS or an equivalent concentration of DMSO in PBS (vehicle solution for all non-treated mice). Body weight was monitored for 5 days following injection. At either 1 or 5 days after injection, mice were euthanized under isoflurane anesthesia followed by cervical dislocation for collection of liver tissue. Fresh liver tissue was used for biochemical assays and molecular analyses, while fixed liver sections were prepared for histological studies as described below.

For the gain-of-function study, Ad-CMV-hStarD5.MycDDK or control Ad-CMV-β-gal were administered to StarD5^−/−^ mice (10-week-old males) via retro-orbital vein injection (1 × 10^11^ genome copies per mouse). Expression of hStarD5 post-injection was detected after 2 days, reaching a peak around 5 days, and remaining stable for up to 2 weeks. At 7 days post-injection, the mice were injected intraperitoneally with tunicamycin at 0.1, 0.25, or 0.5 mg/kg diluted in sterile PBS. Mice were euthanized 5 days after tunicamycin administration, as described above.

### 4.3. Histological Analysis

Unfrozen liver sections were fixed in 4% paraformaldehyde in PBS for 48 h, embedded in paraffin, sectioned (10 μm), and stained with hematoxylin and eosin (H&E) to evaluate gross morphology.

For detection of StarD5, liver sections were deparaffinized in o-xylene and then rehydrated by passage through a graded series of ethanol (100–70%) and distillated water. Antigen retrieval was accomplished with Retrieval Solution (BD Pharmigen, Cat# 550524, La Jolla, CA, USA) in an IHC pressure cooker for 20 min, followed by a 2 h cool-down and washes with water and PBS + 0.05% Tween 20. Hydrogen peroxidase blocking was performed by incubating the slide in a 0.3% H_2_O_2_ solution in PBS for 15 min at room temperature (RT). Samples were then blocked with Avidin D for 15 min at RT and goat serum blocking buffer (1.5% goat serum + 0.03 g BSA in 1 mL of PBS) for 1 h at RT. For interaction with the primary antibody (StarD5 ab), sections were incubated in goat serum blocking buffer with the StarD5 antibody (dilution 1:50) for 16 h at 4 °C in a humidified chamber. The samples were then washed 2 × 5 min in PBS. For the secondary antibody, SignalStain Boost IHC Detection reagent (HRP, Mouse) (Cell Signaling, Cat#8125, Danvers, MA, USA) was used as described by the manufacturer and then developed using a DAB substrate for 5 min. Slides were then rinsed in water three times and stained with hematoxylin for 5 min, and washed under running tap water for an additional 5 min. Finally, the slides were dehydrated by passage through a graded series of ethanol (70–100%) and mounted.

### 4.4. Cholesterol and Triglyceride Quantification

Liver cholesterol and triglyceride quantifications were performed as previously described [18], using the Amplex Red Cholesterol assay kit (Invitrogen, Cat#A12216, Carlsbad, CA, USA) and the Infinity Triglycerides assay kit (Thermo-Fisher Scientific, Cat#22421, Waltham, MA, USA).

### 4.5. Immunoblots

Immunoblotting of liver protein samples was performed as previously described [18], using the antibodies listed in Table 1.

### 4.6. Data Reproducibility and Statistical Analysis

Data are shown as means ± standard deviation (SD) and were analyzed using Prism 10 software (Graphpad, La Jolla, CA, USA). Statistical significance of differences was determined by multiple unpaired, two-tailed Student’s *t*-tests with values *p* ≤ 0.05 considered statistically significant.

## 5. Conclusions

The ability of a cell to shift cholesterol to the plasma membrane while reducing cholesterol content in the ER is viewed as a key element of the cell’s survival response under acute ER stress. We provide evidence for StarD5’s critical role in the mitigation of ER stress through its ability to be induced, and to bind and translocate cholesterol. Additionally, as previously shown [18], ablation of StarD5 impairs VLDL secretion, leading to accumulation of triglycerides in the liver. This may explain the increase in liver triglycerides in the livers of StarD5^−/−^ mice compared to WT mice. It is possible that in conditions such as MAFLD, chronic surplus increases in triglycerides in the liver drive ER stress through a pathway of insulin resistance, as previously described [24,25]. Therefore, StarD5 may also mitigate ER stress through its ability to maintain lipid homeostasis, leading to restoration of normal proteostasis and therefore protecting the liver from MAFLD progression. Thus, maintaining the expression levels of StarD5 may represent a possible therapeutic target for NASH and MAFLD, given its role in lipid homeostasis and fibrosis; its downregulation or ablation has been shown to accelerate the progression of steatosis and liver fibrosis [18].

## Figures and Tables

**Table 1:** Antibodies used for immunoblotting.

Antibody	Company Name (Reference)	Dilution
Anti-HMGCR	Abcam (ab174830)	1:1000
Anti-FLAG	Sigma (F7425)	1:4000
Anti-PARP	Cell Signaling (9542)	1:1000
Anti-FAS	Abcam (ab22759)	1:1000
Anti-StarD5	Santa Cruz (sc-514236)	1:400
Anti-GRP78/BiP	Abcam (ab21865)	1:3000
Anti-GAPDH	Cell Signaling (2118S)	1:2000
Anti-β-actin	Sigma (A5441)	1:3000
Goat anti-rabbit HRP-conjugated IgG	BioRad (170-6515)	1:2000
Goat anti-mouse HRP-conjugated IgG	BioRad (170-6516)	1:2000

## Data Availability

The original contributions presented in this study are included in the article and Supplementary Materials. Further inquiries can be directed to the corresponding author.

## Supplementary Materials

The following supporting information can be downloaded at: https://www.mdpi.com/article/10.3390/ijms26094157/s1.

## Abbreviations

The following abbreviations are used in this manuscript:

ATF6	activating transcription factor 6
ER	endoplasmic reticulum
FAS	fatty acid synthase
GRP78/BiP	glucose-regulated protein 78
HMG-CoA	3-hydroxy-3-methylglutaryl coenzyme A
HRP	horseradish peroxidase
IRE	inositol-requiring enzyme I
PARP	poly (ADP-ribose) polymerase
PERK	protein kinase-like ER kinase
PM	plasma membrane
StarD	steroidogenic acute regulatory-related lipid transfer domain
TG	Triglyceride
Tm	Tunicamycin
UPR	unfolded protein response
WD	Western diet
WT	wild type
