# Supplementary figures and images for: StarD5 Plays a Critical Role in the Hepatocyte ER Stress Survival Response
Source: Int J Mol Sci. 2025 Apr 27;26(9):4157. doi: 10.3390/ijms26094157 (PMC12072137; doi:10.3390/ijms26094157)

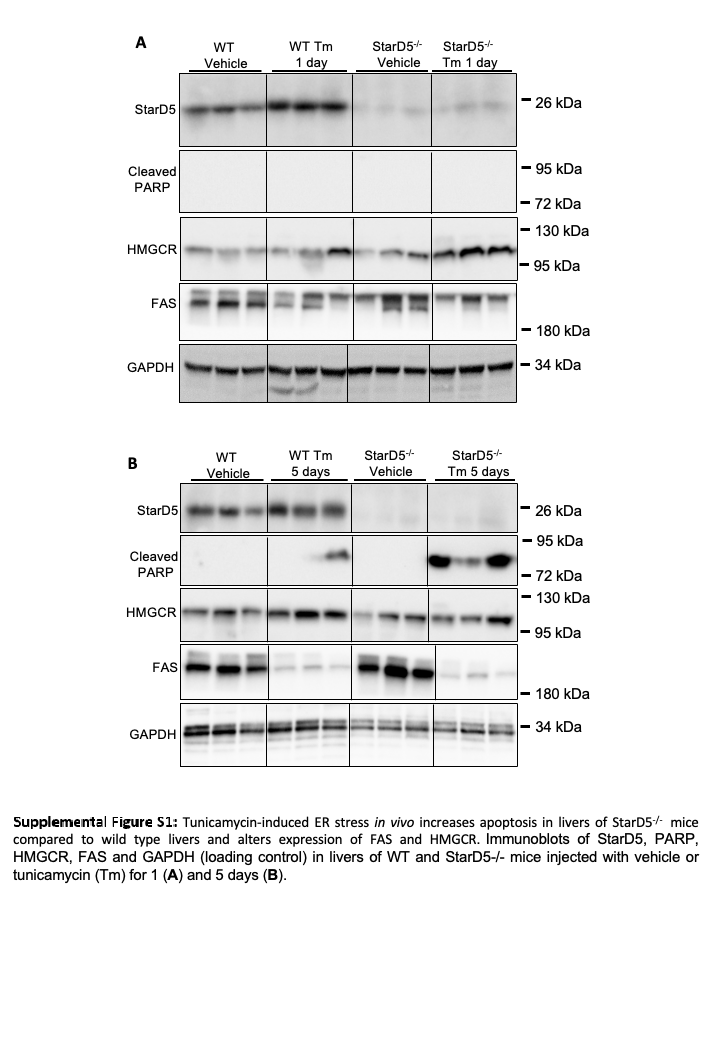

Supplement: Supplementary file 1 [file ijms-26-04157-s001.zip › ijms-3554027-supplementary.tiff]
